# Alkaloid extract of Corydalis yanhusuo inhibits angiogenesis via targeting vascular endothelial growth factor receptor signaling

**DOI:** 10.1186/s12906-019-2739-6

**Published:** 2019-12-10

**Authors:** Li Wan, Yang Zhao, Qun Zhang, Guangyi Gao, Shanlan Zhang, Yong Gao, Xiaofei Chen, Xiaoping Qian

**Affiliations:** 1 Comprehensive Cancer Center, Nanjing Drum Tower Hospital Clinical College of Traditional Chinese and Western Medicine, Nanjing University of Chinese Medicine, Nanjing, 210008 China; 2 Department of Clinical Oncology, Huai’an First People’s Hospital, Nanjing Medical University, Huai’an, 223300 China; 3 School of Pharmacy, Nanjing University of Chinese Medicine, Nanjing, 210023 China; 4 Comprehensive Cancer Center, Nanjing Drum Tower Hospital, Medical School of Nanjing University, Clinical Cancer Institute, Nanjing University, Nanjing, 210008 China; 5 Department of Traditional Chinese Medicine, Huai’an Second People’s Hospital, The Affiliated Huai’an Hospital of Xuzhou Medical University, Huai’an, 223000 China; 6 Department of Oncology, Songjiang District Central Hospital, Shanghai, 201600 China

**Keywords:** Corydalis yanhusuo, Alkaloid extract, Tube formation, Sprouting, Angiogenesis, Vascular endothelial growth factor receptor, AKT signaling

## Abstract

**Background:**

*Corydalis yanhusuo* W.T. Wang (YHS) is a well-known Chinese flowering herbal plant commonly used for centuries in functional food and traditional Chinese medicine. In the present study, we have identified and characterized a novel inhibitor of vascular endothelial growth factor receptor 2 (VEGFR2) with low toxicity, alkaloid extract of YHS, which suppressed angiogenesis that plays a fundamental role in a wide spectrum of physiological functions and pathological processes.

**Methods:**

Proliferative ability of human umbilical vascular endothelial cells (HUVECs) was assessed using MTT assay and Ki67 immunofluorescence staining. Migration ability of HUVECs was evaluated by wound healing and transwell assays. In vitro angiogenesis was tested by spheroid sprouting and tube formation assays. In vivo vascularization was examined using Matrigel plug and chick chorioallantoic membrane (CAM) models. Protein expression and phosphorylation levels of VEGFR2, AKT, ERK and STAT3 were determined by Western blot assay.

**Results:**

We demonstrated that alkaloid extract of YHS significantly inhibited a variety of VEGF-induced angiogenesis processes including proliferation, migration, sprouting, and tube formation of HUVECs. Moreover, alkaloid extract of YHS contributed to reduced in vivo neo-vessel formation in Matrigel plugs of mice and CAM models. Further mechanistic studies revealed that alkaloid extract of YHS suppressed VEGF-induced signaling pathway as evaluated by diminished phosphorylation of VEGFR2 and subsequently attenuated its downstream regulators including phospho-ERK1/2, phospho-AKT and phospho-STAT3 levels in HUVECs.

**Conclusion:**

Collectively, these preclinical findings indicate that alkaloid extract of YHS remarkably limits angiogenesis and may serve as a promising anti-angiogenic drug candidate.

## Background

Angiogenesis, the formation of new vessels from pre-existing blood vessels and the modeling of the newly formed vascular network, has been well recognized as a fundamental requirement to facilitate tumor progression and to support various ischemic and inflammatory diseases [1–3]. Angiogenesis appears to be a highly dynamic and complicated multifaceted process, which is closely associated with the function of endothelial cells, such as the activation, adhesion, proliferation and transmigration of endothelial cells from blood vessels, the establishment of suitable connections, tube formation, remodelling and pruning, and the recruitment of mural cells [4, 5]. Tumor angiogenesis starts with malignant tumor cells releasing molecules that transmit signals to surrounding normal tissue. These signals activate certain genes in host endothelial cells that, in turn, produce proteins to promote formation and growth of new blood vessels [6]. Therefore, the growth and spread of tumors can be limited by inhibiting angiogenesis, and numerous studies have illustrated this through the use of antiangiogenic drugs to starve the tumor cells of nutrients [7].

Vascular endothelial growth factor (VEGF) is known as one of the most fundamental regulators of angiogenesis. It is a growth and survival factor for endothelial cells (ECs) and a critical mediator of angiogenesis via VEGF receptor 2 (VEGFR2)-dependent signaling pathway [8]. VEGFR2 as the major effector for execution of VEGF signaling is composed of three different parts, including an extracellular domain with seven immunoglobulin-like repeats, a hydrophobic transmembrane region containing the tyrosine kinase domain and the carboxyl terminal tail [9]. A growing body of evidence has shown that VEGF binds to VEGFR2 and then turns on the intracellular signaling cascades involving the PKC-MEK-ERK1/2, PI3K-AKT-mTOR and JAK2-STAT3 pathways to contribute to endothelial cell migration, proliferation and survival [10]. VEGFR2 is also able to interact with the β1 integrin in a matrix-bound VEGF-dependent manner, which results in prolonged phosphorylation of p38 mitogen-activated protein kinase (MAPK) and association of β1 integrin with focal adhesions [11]. Notably, VEGFR2-PI3K-induced membrane-bound PIP_3_ production triggers phosphorylation of PKB/AKT by phosphoinositide-dependent kinases 1 and 2 (PDK1 and PDK2), AKT phosphorylates BCL-2 associated death promoter (BAD) and caspase 9 and thus inhibits their apoptotic activity [8, 12]. More importantly, VEGF stimulation of cultured endothelial cells induced STAT3 phosphorylation by a VEGFR2-dependent mechanism. STAT3 as a transcriptional factor translocates to the nucleus and binds to nuclear DNA to modulate transcription of target genes, such as BCL-2 and ANG2 that are closely related to angiogenesis [13, 14].

Over the last few decades, emerging evidence suggests that VEGFR2 has become one of effective and efficient therapeutic targets for cancer therapy. A list of orally active small molecular inhibitors of VEGFR2 have been well reported, including various drugs approved by the Food and Drug Administration (FDA) such as sunitinib, sorafenib and vandetanib [15, 16]. Nevertheless, long-term treatment with these agents in clinic might be associated with distinct adverse effects, including bleeding complications [17], hypertensive crisis, and gastrointestinal perforation [18], suggesting that discovery and development of novel VEGFR2 inhibitors with low toxicity are still urgently required. Thus, more attention has been paid to natural products as a promising alternative route in targeting VEGFR2 to prevent tumor angiogenesis.

*Corydalis yanhusuo* W.T. Wang (YHS) is a well-known Chinese flowering herbal plant commonly used for centuries in functional food and traditional Chinese medicine to alleviate pain [19]. Over the past few years, extensive literature has accumulated on that YHS possesses various pharmacological activities. It has been reported that YHS effectively diminishes acute, inflammatory and neuropathic pain at least partially mediated through dopamine D2 receptor antagonism [20]. In addition, YHS attenuates infarct size and enhances heart function during myocardial ischemia/reperfusion by inhibiting apoptosis via regulation of the BCL-2 family in rats [21]. Furthermore, YHS was also found to exert the anti-proliferative effects on MCF-7 breast cancer cells by inducing cell cycle G2/M arrest [22] and lead to decreased migration and invasion of MDA-MB-231 breast cancer cells involved the inhibition of MAPK signalling [23]. The alkaloid components are considered as the main bioactive ingredients of YHS. It has been shown that the alkaloid ingredients of YHS including tetrahydropalmatine are essential for inhibiting cytochromes P450 (CYPs) activity in vitro [24].

In the present study, we have illustrated that alkaloid extract of YHS exerted striking anti-angiogenesis effects both in vitro and in vivo*,* which was particularly reflected by a serious of biological behaviors of human umbilical vein endothelial cells (HUVECs) and various angiogenesis models. In light of the underlying mechanisms, the inhibitory effects of alkaloid extract of YHS on angiogenesis were related to the suppression of VEGFR2 activation and its downstream AKT, ERK and STAT3 signaling transduction. To this end, our results imply that YHS is able to act as an effective natural VEGFR2 inhibitor that may be further developed to be a therapeutic agent for angiogenesis-associated diseases.

## Methods

### Materials and reagents

3-(4,5-dimethylthiazol-2-yl)-2,5-diphenyltetrazolium (MTT) and dimethyl sulfoxide (DMSO) were obtained from Sigma-Aldrich (St. Louis, MO). Recombinant human (Cat. No. 293-VE/CF) and mouse VEGF (Cat. No. 7916-MV) were both purchased from R&D Systems. Growth factor-reduced phenol red-free Matrigel (Cat. No. 356237) was from BD Biosciences (Bedford, MA). Lactate dehydrogenase (LDH) kit (Cat. No. A020–2) was purchased from the Nanjing Jiancheng Bioengineering Institute (Nanjing, China). Most appropriate primary antibodies as well as the corresponding secondary antibodies used in this study were obtained from Cell Signaling Technology (Beverly, MA).

### Drug preparation

YHS was purchased from Nanjing Hospital of Traditional Chinese Medicine (Cat. No. 110116). The alkaloid fractions of YHS were extracted in the lab by using a general method as previously described [25]. Briefly, 100 g of whole dry root of Corydalis yanhusuo was ground with a homogenizer and then extracted three times with 2.5 L of 60% ethanol for 1 h in an ultrasonic bath. The extracts were combined and filtrated under vacuum, followed by evaporated to dryness with rotary vaporization under reduced pressure. The residue oily solid was suspended in 1.5 L of 0.01 M hydrochloric acid. After filtration, the pH of the solution was adjusted to 12 with NaOH, followed by extracted with 1.5 L of ethyl acetate three times. The ethyl acetate fractions were combined and concentrated to dryness with rotary vaporization at 60 °C under reduced pressure. The total alkaloids extract was dissolved in DMSO and filtered with 0.22 μm membrane. The final concentrations of DMSO for different treated groups were 2% (100 μg/ml alkaloid extract of YHS), 1% (50 μg/ml alkaloid extract of YHS) and 0.2% (10 μg/ml alkaloid extract of YHS). In addition, the final concentration of DMSO for vehicle control group was 2% to keep consistence with that of highest drug concentration group.

### Cell culture

HUVECs were isolated from human umbilical cords (obtained from Department of Gynaecology and Obstetrics in Nanjing Drum Tower Hospital, Nanjing, China) by collagenase digestion and grown in M199 media (Sigma-Aldrich) supplemented with 15 μg/ml endothelial cell growth factor (BD Biosciences, MA), 15 μg/ml heparin (Sigma-Aldrich) and 15% fetal calf serum (FCS) at 37 °C under 5% CO_2_ as previously described [26]. Confluent HUVECs were routinely passaged by trypsinization and utilized for experiments at passages 2–4.

### Cell viability assay

In brief, 1 × 10^4^ HUVECs per well were plated into gelatin (0.1%) coated 96-well tissue culture plates for overnight attachment, and then the cultivated medium was replaced with serum-free medium supplemented with various concentrations of alkaloid extract of YHS (2.5–200 μg/ml) in the absence or presence of VEGF (20 ng/ml) in a final volume of 200 μl/well. After 24 h. Cell viability was evaluated by MTT assay and three independent experiments with triplicate were performed.

### LDH toxicity assay

This experiment was performed according to the manufacturer’s instructions. Briefly, 5 × 10^3^ HUVECs per well were seeded in 96-well plates and incubated at 37 °C and 5% CO_2_ for overnight attachment. The HUVECs were then incubated in the presence of various concentrations of alkaloid extract of YHS (2.5–200 μg/ml) as indicated for 24 h. Cell supernatants were then collected from each well and analysed for LDH cytotoxicity. The absorbance of formed formazan was read through at 490 nm on a microplate reader.

### Cell migration

The migration of HUVECs was evaluated by wound-healing and transwell migration assays as described previously [27]. In terms of wound-healing assay, 5 × 10^5^ HUVECs per well were plated into a six-well plate coated with gelatin. Upon reaching about 90–95% confluence, the cell monolayer was carefully scraped by a sterile 1 ml pipette tip to create wounds (t = 0 h). Cellular debris was removed by washing with PBS and the media was replaced with serum-free medium containing different concentrations of alkaloid extract of YHS with 20 ng/ml VEGF. Following 12 h and 24 h of incubation, HUVECs were photographed with an inverted bright field microscopy and the gap distance was quantified manually using Fiji software. For transwell assay, 200 μl HUVECs at a density of 5 × 10^5^/ml were plated into the upper chamber of transwell (Costar) with 8.0 μm pore polycarbonate insert and 600 μl of HUVEC medium with 20 ng/ml VEGF was added in the bottom chamber, various concentrations of alkaloid extract of YHS were then added to both chambers. The cells in the upper chamber were removed after 7 h of incubation, and the migrated HUVECs were fixed in 4% paraformaldehyde (PFA) and stained with 0.1% crystal violet for 30 min. Images were taken with an inverted microscopy and migration of HUVECs was quantified.

### Spheroid sprouting assay

Spheroid sprouting assay was carried out as described previously with minor modifications [28]. In brief, HUVECs treated with various concentrations of alkaloid extract of YHS for 24 h were suspended in HUVEC medium with 20% methylcellulose and seeded into U-bottom 96-well plates in a final density of 6000 cells/ml to form spheroids. The spheroids were then collected and overlayed with methylcellulose with 40% FCS and collagen solution consisting of 2 mg/ml rat tail collagen (BD Biosciences), EBSS and 20 mM NaOH. 800 μl of the spheroids/collagen mix were plated to a 24-well plate to incubate at 37 °C for 30 min. 200 μl HUVEC medium containing 25 ng VEGF was used to stimulate spheroids formation for 24 h. Images were taken with an inverted bright field microscopy. The length and total number of sprouts from at least 20 fields were manually quantified with Fiji software.

### Tube formation assay

Tube formation assay was performed as previously described [16]. Briefly, HUVECs (3.5 × 10^4^/well) treated with various concentrations of alkaloid extract of YHS for 24 h were seeded into a 96-well plate precoated with 100 μl Matrigel. Angiogenesis was assessed on the basis of capillary-like structure formation after 7 h treatment. Images were taken with an inverted bright field microscopy. Tube length was measured by using Fiji software and the result was normalized to control.

### Immunofluorescence staining

HUVEC Immunofluorescence staining was performed as previously described with minor modifications [29]. HUVECs at a density of 5 × 10^4^/well treated with various concentrations of alkaloid extract of YHS for 24 h were plated into Lab-Teck chamber slides (Thermo Fisher Scientific) coated with fibronectin. After overnight incubation, the cells were fixed with 4% PFA and permeabilized with 0.1% Triton X-100 in PBS containing 5% bovine serum albumin. HUVECs were then incubated at 4 °C overnight with the primary antibody, followed by 1 h of incubation with a secondary antibody at room temperature in the dark. Nuclei were counterstained with DAPI and images were taken with a confocal microscopy (Leica TCS SP5).

### Western blot

HUVECs treated with different concentrations of alkaloid extract of YHS for 24 h were starved in serum-free medium for 2 h and then stimulated with 20 ng/ml of VEGF. Protein extraction, separation and transfer were carried out according to the established protocol [30]. The PVDF membranes were blocked with 5% skim milk powder and probed overnight at 4 °C with indicated primary antibodies. HRP-conjugated secondary antibodies were incubated for 2 h at room temperature after washing with PBS containing 0.1% Tween-20. The chemiluminescent signals were detected using the enhanced chemiluminescence (ECL) reagent. β-actin was used as a loading control.

### Chick embryo chorioallantoic membrane (CAM) assay

CAM assay was based on previously described method with minor modifications [31]. In brief, at day 3 of post incubation, 2 ml of albumin were taken off and a square window (around 1 cm^2^) in the egg shell was created, followed by sealing with paraffin film to prevent dehydration. The eggs were incubated for the next five days and then treated with various concentrations of alkaloid extract of YHS with 20 ng/ml VEGF for 48 h at which vascularization potential of the CAM reached its maximum. Control and treated CAM specimens were photographed using digital camera.

### Matrigel plug assay

Mouse studies were reviewed and approved by the Institutional Animal Care and Use Committee of Nanjing Drum Tower Hospital and performed in accordance with the guidelines of the Institutional Animal Care and Use Committee of Nanjing Drum Tower Hospital. Matrigel plug assay was performed as previously described with minor modifications. 500 μl Matrigel supplemented with 50 ng VEGF was injected subcutaneously into the right flank of C57BL/6 mice (8–12 weeks old). Different concentrations of alkaloid extract of YHS (0 mg/kg, 15 mg/kg, 30 mg/kg and 60 mg/kg) were intraperitoneally injected into mice twice per week. The mice were euthanized by inhalation of CO_2_ in a euthanizing chamber 14 days following implantation. Matrigel plugs were harvested and then fixed, processed, embedded in wax and cut into 4 μm sections on a microtome. The sections of Matrigel plugs were stained with CD31 antibody and vessel number was quantified by Fiji software.

### Statistical analysis

Statistical analysis was carried out using GraphPad Prism version 7.0 (GraphPad Software, San Diego, USA). All experiments were performed in triplicates and the quantitative data are presented as mean ± SEM. Comparisons were conducted by two-tailed Student’s t-test and *P* value < 0.05 was considered to indicate statistical significance.

## Results

### Alkaloid extract of YHS suppressed VEGF-mediated HUVEC proliferation

**Fig. 1:**
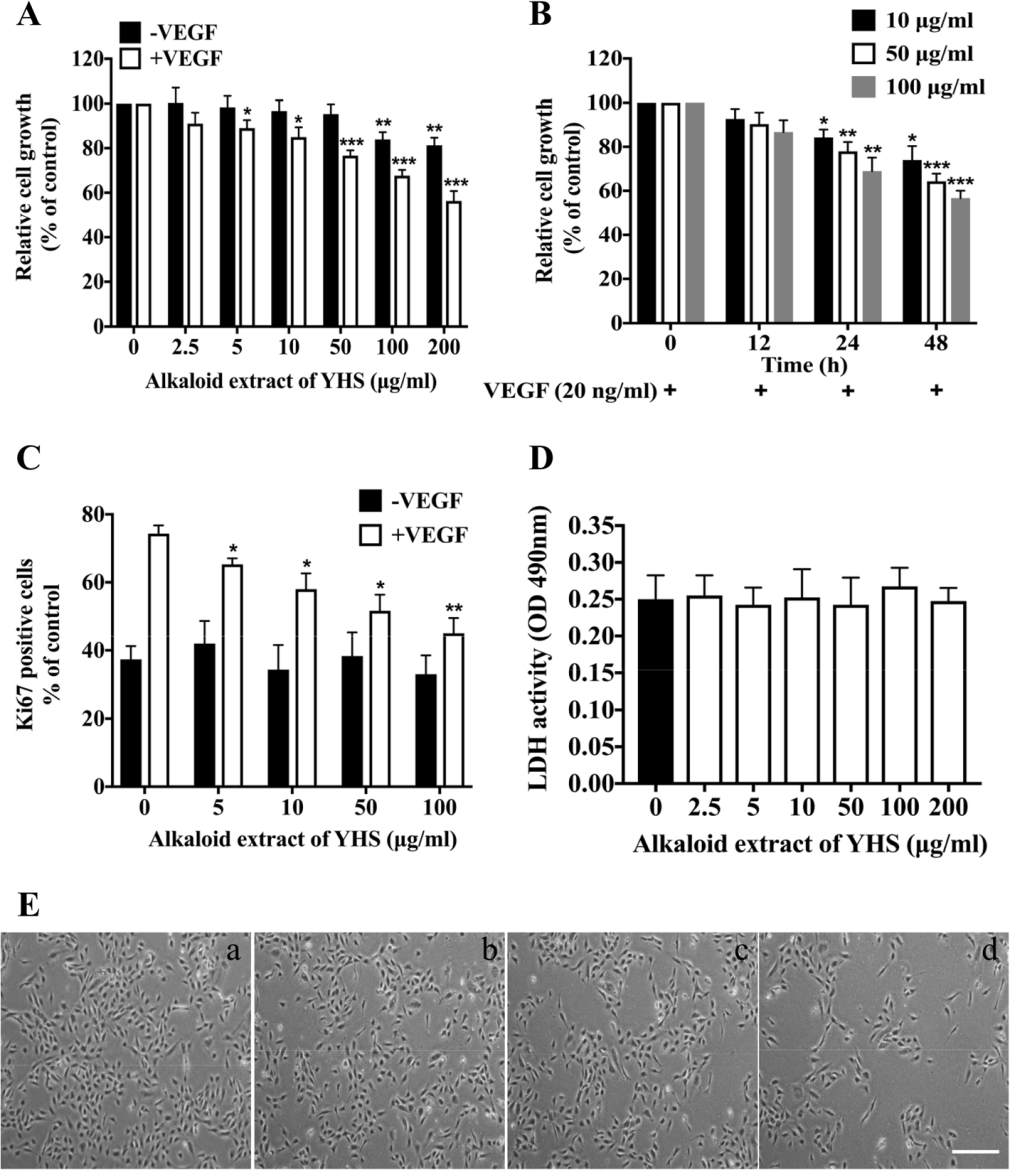
Alkaloid extract of YHS suppressed VEGF-mediated HUVEC proliferation (**a**) The proliferation of HUVECs cultured in standard media after the treatment of various concentrations of alkaloid extract of YHS for 24 h with and without VEGF. Data are presented as mean ± SEM. ***, *P* < 0.001; **, *P* < 0.01; *, *P* < 0.05, paired t-test. **b** Time-course study of alkaloid extract of YHS on proliferation of HUVECs. HUVECs were treated with various concentrations of alkaloid extract of YHS for 12, 24 and 48 h in the presence of VEGF, after which HUVEC proliferation was detected by MTT assay. Data are presented as mean ± SEM. ***, *P* < 0.001; **, *P* < 0.01; *, *P* < 0.05, paired t-test. **c** Ki67 staining in HUVECs that had been treated with various concentrations of alkaloid extract of YHS for 24 h in the absence and presence of VEGF. Proliferation was determined by the ratio of the average number of Ki67-positive cells to total cells. Data are presented as mean ± SEM. **, *P* < 0.01; *, *P* < 0.05, paired t-test. **d** LDH release from HUVECs after the treatment of various concentrations of alkaloid extract of YHS for 24 h. Data are presented as mean ± SEM, paired t-test. **e** Representative images of HUVEC morphology after 48 h exposure to (**a**) 0 μg/ml; (**b**) 10 μg/ml; (**c**) 50 μg/ml; (**d**) 100 μg/ml of alkaloid extract of YHS in the presence of 20 ng/ml VEGF, scale bar: 250 μm

In order to systematically determine the anti-angiogenic activity of alkaloid extract of YHS in vitro, we first examined its effects on VEGF-mediated HUVEC proliferation. As shown in Fig. 1a, the proliferation of HUVECs stimulated with VEGF was dramatically attenuated after the treatment of alkaloid extract of YHS ranging from 5 to 200 μg/ml. Surprisingly, alkaloid extract of YHS had slightly weaker inhibitory effects on the proliferation of HUVECs in the absence of VEGF though it still significantly decreased HUVEC proliferation at the concentrations of 100 μg/ml and 200 μg/ml. In addition, HUVECs were treated with alkaloid extract of YHS at different time intervals of 12, 24, and 48 h to figure out the time course of drug effects. It was revealed that alkaloid extract of YHS ranging from 10 to 100 μg/ml inhibited the proliferation of HUVECs induced by VEGF in a time-dependent manner and there were significant differences at 24 and 48 h compared to 0 h treatment (Fig. 1b). In agreement with MTT assay, immunofluorescence staining of HUVECs stimulated with or without VEGF using the proliferative marker Ki67 revealed that alkaloid extract of YHS dose-dependently lowered the proliferative capability of HUVECs in the presence of VEGF but exhibited no obvious effects in the absence of VEGF (Fig. 1c). To evaluate whether decreased proliferation by alkaloid extract of YHS was due to its toxicity effects on HUVECs, LDH cytotoxicity assay was performed. As illustrated in Fig. 1d, no toxic effect on HUVECs was detected after the treatment of alkaloid extract of YHS (from 2.5 to 200 μg/ml) compared to vehicle control. In line with LDH assay, HUVEC morphology was found to be normal and exhibited typical ‘cobblestone’ appearance following the treatment of various concentrations of alkaloid extract of YHS (Fig. 1e), indicating the low toxicity of alkaloid extract of YHS to HUVECs.

### Alkaloid extract of YHS inhibited migration, sprouting and tube formation of HUVECs

**Fig. 2:**
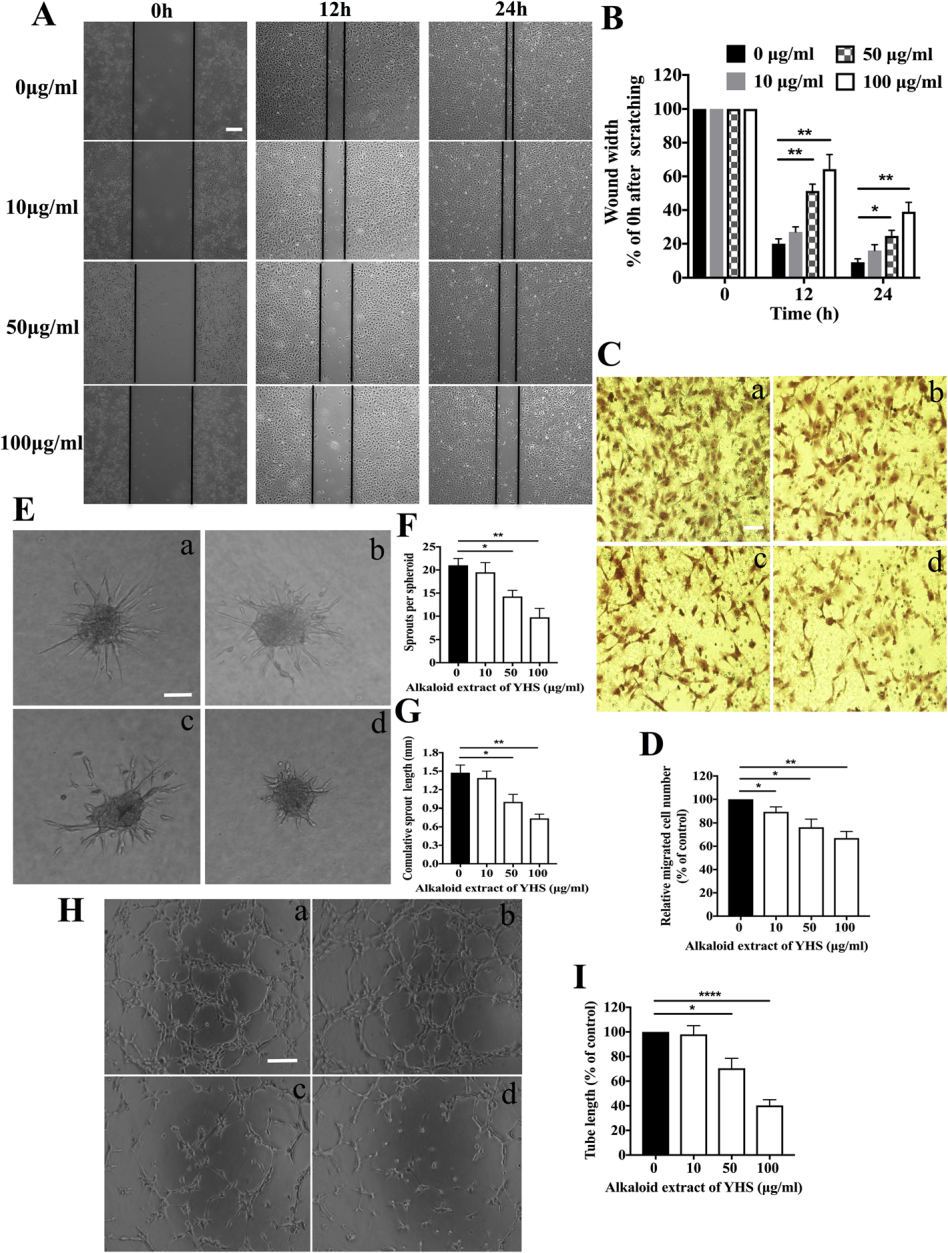
Alkaloid extract of YHS inhibited migration, sprouting and tube formation of HUVECs (**a**) Representative images from a time-lapse sequence (0, 12 and 24 h) of HUVECs migrating to heal a wound scratched in a monolayer following the treatment of various concentrations of alkaloid extract of YHS in the presence of VEGF, scale bar: 250 μm. **b** Quantification of HUVEC horizontal migration. The migration of cells toward the wounds represents percentage of wound width. Wound width at time zero (t = 0 h) was set as 100%. Data are presented as mean ± SEM. **, *P* < 0.01; *, *P* < 0.05, paired t-test. **c** Representative images of vertical migration of HUVECs after exposure to (**a**) 0 μg/ml; (**b**) 10 μg/ml; (**c**) 50 μg/ml; (**d**) 100 μg/ml of alkaloid extract of YHS. Migrated cells stained with crystal violet were imaged with an inverted bright field microscopy, scale bar: 100 μm. **d** Quantification of HUVEC vertical migration by quantifying the number of migrated cells. Relative migrated cell number of HUVECs treated with 0 μg/ml alkaloid extract of YHS was set as 100%. Data are presented as mean ± SEM. **, *P* < 0.01; *, *P* < 0.05, paired t-test. **e** Representative images of spheroid formed by HUVECs after exposure to (**a**) 0 μg/ml; (**b**) 10 μg/ml; (**c**) 50 μg/ml; (**d**) 100 μg/ml of alkaloid extract of YHS in the presence of VEGF, scale bar: 100 μm. **f** Quantitative analysis of the number of sprouts. Data are presented as mean ± SEM. **, *P* < 0.01; *, *P* < 0.05, paired t-test. **g** Quantification of cumulative sprouts length. Data are presented as mean ± SEM. **, *P* < 0.01; *, *P* < 0.05, paired t-test. **h** Representative images of HUVEC tube formation after exposure to (**a**) 0 μg/ml; (**b**) 10 μg/ml; (**c**) 50 μg/ml; (**d**) 100 μg/ml of alkaloid extract of YHS, scale bar: 100 μm. **i** Quantitative analysis of tube length. Relative tube length of HUVECs treated with 0 μg/ml alkaloid extract of YHS was set as 100%. Data are presented as mean ± SEM. ****, *P* < 0.0001; *, *P* < 0.05, paired t-test

Considering the critical role of endothelial cell motility in promoting angiogenesis, we further explored the potential function of alkaloid extract of YHS in modulating the migration of HUVECs. To this end, wound-healing and transwell assays were carried out to assess the horizontal and vertical migration capabilities of HUVECs, respectively. As illustrated in Fig. 2a and b, the gap distances between one side of scratch of alkaloid extract of YHS at the concentrations of 10, 50 and 100 μg/ml were much wider than that of vehicle control at 12 and 24 h, implying that alkaloid extract of YHS could concentration-dependently diminish the migration capability of HUVECs. This was also substantiated by the transwell migration assay that used for assessing cell vertical migration ability, which demonstrated that less HUVECs treated with different concentrations of alkaloid extract of YHS migrated to the lower chamber compared to vehicle control after 7 h of incubation (Fig. 2c and d).

Alternative routes to evaluate drug efficacy against angiogenesis include the sprouting and tube formation assays, which are able to reflect the migration, intercellular interactions and differentiation of endothelial cells. In terms of sprouting assay that is recognized as the initial step of angiogenesis, it was found that alkaloid extract of YHS lead to significant reductions in both the number of sprouts and the cumulative sprout length of the spheroids in a dose-dependent manner, and there were significant differences in endothelial cell sprouting from 50 μg/ml alkaloid extract of YHS compared to vehicle control (Fig. 2e-g). To further confirm the inhibitory role of alkaloid extract of YHS in angiogenesis, tube formation assay of HUVECs was conducted accordingly. It was shown that VEGF treatment on HUVECs facilitated perfect formation of capillary-like network. However, the capillary-like tubular structure on Matrigel after the treatment of alkaloid extract of YHS ranging from 10 to 100 μg/ml was dose-dependently decreased in the presence of VEGF and there were significant differences in terms of tube length with 50 and 100 μg/ml alkaloid extract of YHS treatment compared to vehicle control (Fig. 2h and i).

### Alkaloid extract of YHS limited angiogenesis in vivo

**Fig. 3:**
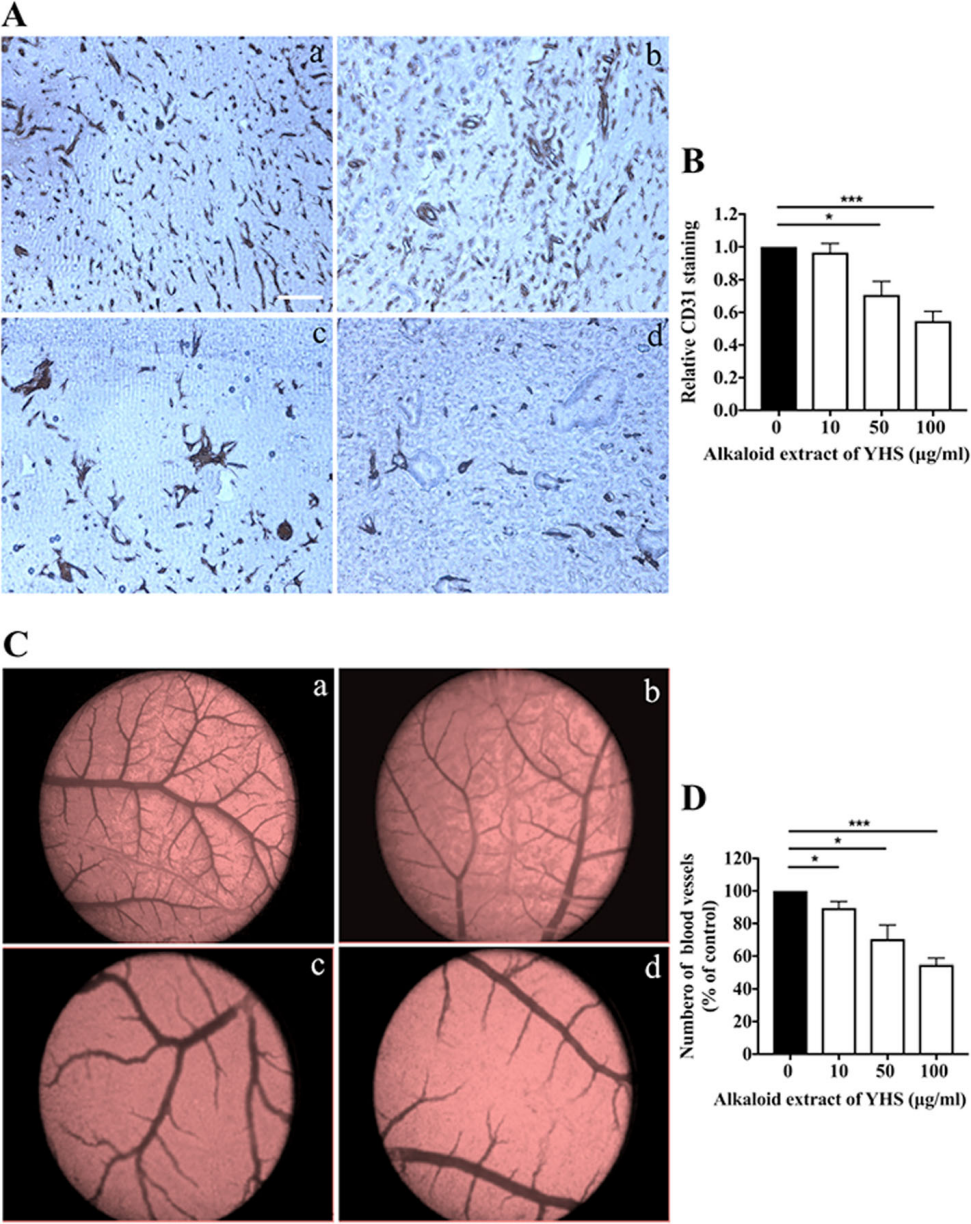
Alkaloid extract of YHS limited angiogenesis in vivo (**a**) Antiangiogenic effects of alkaloid extract of YHS in mice. C57BL/6 Mice were subcutaneously injected with 500 μl of a Matrigel mixture containing 50 ng VEGF. The mice treated with various concentrations of alkaloid extract of YHS (a: 0 mg/kg; b: 15 mg/kg; c: 30 mg/kg; d: 60 mg/kg) were euthanized 14 days later and Matrigel plugs were harvested. 4 μm paraffin sections were stained with CD31 (brown) and a representative image is shown, scale bar: 200 μm. **b** Quantification of relative vessel area (CD31 positive staining). Relative CD31 positive staining treated with 0 mg/kg alkaloid extract of YHS was set as 100%. Data are presented as mean ± SEM. ***, *P* < 0.001; *, *P* < 0.05, *n* = 4, paired t-test. **c** Representative images of microvessels formation on in vivo CAM model after the treatment of different concentrations of alkaloid extract of YHS (a: 0 μg/ml; b: 10 μg/ml; c: 50 μg/ml; d: 100 μg/ml). **d** Quantification of number of blood vessels in CAM model. The index was defined as the mean number of visible microvessel with the defined area of drug-containing pellets. Relative blood vessel number treated with 0 μg/ml alkaloid extract of YHS (control) was set as 100%. Data are presented as mean ± SEM. ***, *P* < 0.001; *, *P* < 0.05, *n* = 4, paired t-test

To further clarify the prominent anti-angiogenesis effects of alkaloid extract of YHS, Matrigel plug assay in mice was also conducted. The Matrigel plug assay was utilized to evaluate the extent of endothelial cells that had penetrated into the Matrigel plug. The C57BL/6 mice were injected with Matrigel subcutaneously in the presence or absence of VEGF, followed by intraperitoneal injection of various concentrations of alkaloid extract of YHS. The Matrigel plugs were harvested, fixed and processed 14 days following injection. 4 μm paraffin sections were cut and the new blood vessel formation was then examined with immunohistochemistry staining of CD31 that is regarded as a specific endothelial cells marker. Consistent with previous reports, Matrigel plug in the absence of VEGF failed to exhibit obvious vascularization or vascular structure (data not shown). Nevertheless, plugs supplemented with VEGF showed extensive vessels while alkaloid extract of YHS ranging from 15 to 60 mg/kg substantially decreased vascularization of plugs in a dose-dependent manner, which was validated by the CD31 positive staining area (Fig. 3a, b and Table 1).

**Table 1:** The percent inhibition of angiogenesis (Mean ± SEM, %)

Matrigel plug model			CAM model		
10 μg/ml	50 μg/ml	100 μg/ml	10 μg/ml	50 μg/ml	100 μg/ml
3.5 ± 5.5	29.2 ± 8.2*	45.3 ± 5.9***	10.5 ± 4*	29.5 ± 8.5*	45.3 ± 3.9***

Alkaloid extract of YHS limited angiogenesis in Matrigel plug and CAM models. Data are presented as mean ± SEM. ***, *P*<0.001; *, *P*<0.05, paired t-test

Furthermore, CAM assay has been widely verified to be an appropriate in vivo model containing fundamental mechanisms related to physiological and pathological angiogenesis [32, 33]. Thus, CAM model is an effective tool to allow the detection of the ability of alkaloid extract of YHS as an antiangiogenic drug candidate. As shown in Fig. 3c and d, VEGF alone remarkably elevated the microvessels formation in the CAM model, which was demonstrated by production of massive vessel branches. Nevertheless, the number of blood vessels in CAM gradually attenuated with increasing concentrations of alkaloid extract of YHS (from 10 to 100 μg/ml) accompanied by VEGF stimulation (Table 1).

### Alkaloid extract of YHS blocked VEGFR2 signaling pathway

**Fig. 4:**
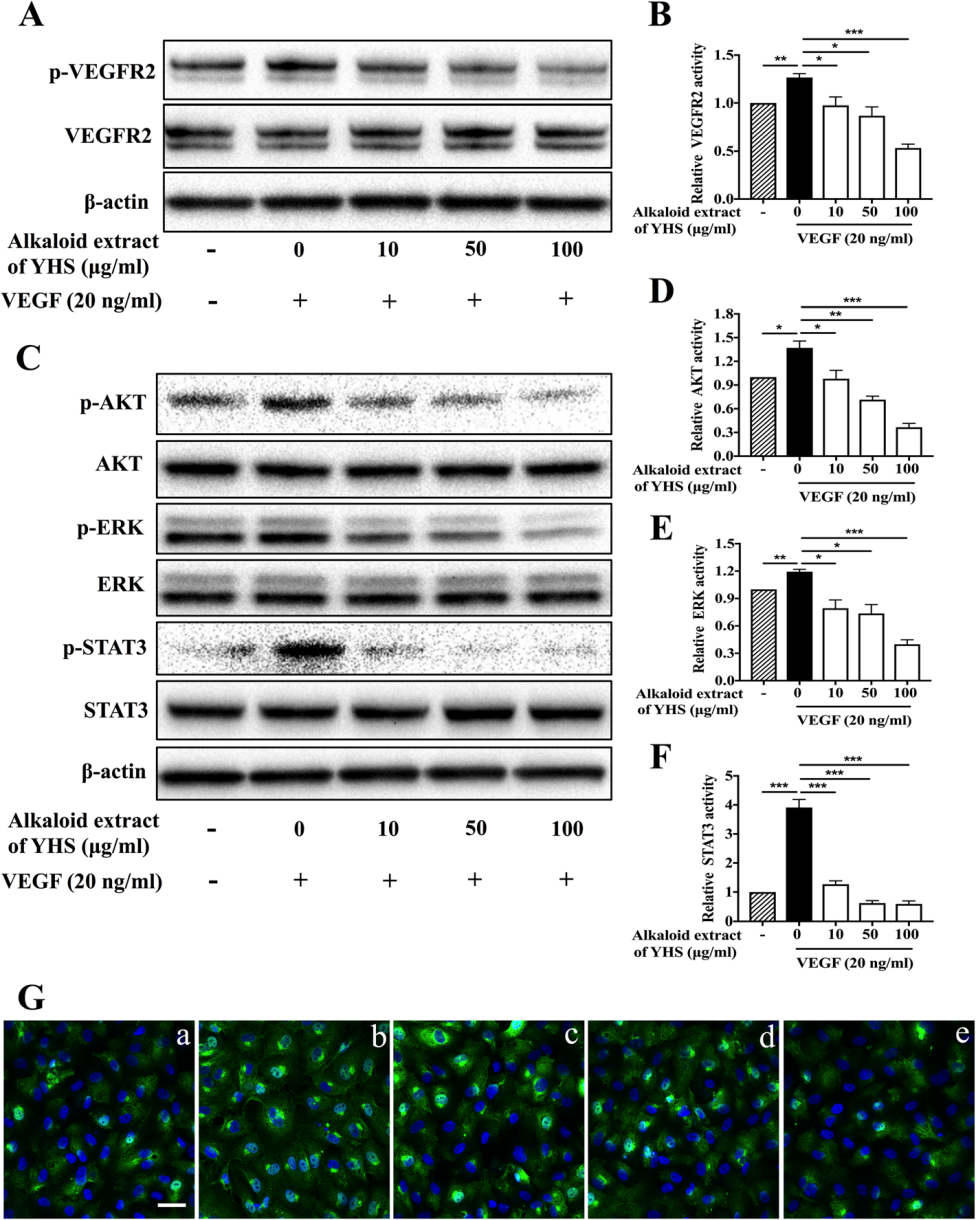
Alkaloid extract of YHS blocked VEGFR2 signaling pathway (**a**) Western blot bands for phosphorylation of VEGFR2 and total VEGFR2 in HUVECs lysates 24 h following various concentrations of alkaloid extract of YHS treatment in the absence or presence of VEGF. β-actin was used as a loading control. **b** Densitometric ratios for VEGFR2 activities were quantified. Data are presented as the normalized expression of mean of three independent HUVEC lines ± SEM. ***, *P* < 0.001; **, *P* < 0.01; *, *P* < 0.05, paired t-test. **c** Western blot bands for phosphorylation of AKT, ERK, STAT3 and total AKT, ERK, STAT3 in HUVECs lysates 24 h following various concentrations of alkaloid extract of YHS treatment in the absence or presence of VEGF. β-actin was used as a loading control. Densitometric ratios for AKT (**d**), ERK (**e**) and STAT3 (**f**) activities were quantified. Data are presented as the normalized expression of mean of three independent HUVEC lines ± SEM. ***, *P* < 0.001; **, *P* < 0.01; *, *P* < 0.05, paired t-test. **g** Immunofluorescence of STAT3 (green) in HUVECs following exposure to (**b**) 0 μg/ml; (**c**) 10 μg/ml; (**d**) 50 μg/ml; (**e**) 100 μg/ml of alkaloid extract of YHS in the presence of VEGF. Translocation of STAT3 in non-treated HUVECs is shown as control (**a**). Nuclei were counterstained with DAPI (blue), scale bar: 50 μm

In order to figure out the underlying mechanisms of alkaloid extract of YHS in exerting anti-angiogenic effect, we thus investigated the signaling pathways mediated by alkaloid extract of YHS in HUVECs using western blot analysis. Given the fact that blockage of VEGFR2 could dramatically prevent angiogenesis process, we first determined the impacts of alkaloid extract of YHS on tyrosine phosphorylation of VEGFR2 (the active form of VEGFR2) stimulated by VEGF. Hence, the total expression and degree of phosphorylation of VEGFR2 (Tyr 1175) were detected with their specific antibodies in the presence of VEGF. As shown in Fig. 4a, VEGFR2 phosphorylation apparently elevated in response to VEGF stimulation. However, alkaloid extract of YHS inhibited VEGF-induced tyrosine phosphorylation of VEGFR2 (Tyr1175) in a dose-dependent manner, while the total levels of VEGFR-2 had little changes. Quantitative densitometry of VEGFR2 phosphorylation was shown as percentage (%) of vehicle control and statistically significant differences were noticed between 0 μg/ml and 10, 50, 100 μg/ml alkaloid extract of YHS (Fig. 4b).

To further confirm that the molecular mechanism of alkaloid extract of YHS on anti-angiogenic property was resulted from blocked VEGF/VEGFR2 signaling, we thus determined its effects on VEGFR2 downstream signaling. Notably, alkaloid extract of YHS dose-dependently suppressed the activation of AKT that is known to be classic VEGFR2 downstream signaling molecule by decreasing its phosphorylation rather than affecting total AKT level (Fig. 4c and d). Moreover, phosphorylation of ERK was diminished following the treatment of alkaloid extract of YHS in a dose-dependent manner though there were no obvious differences in the total expression of ERK between control and treated groups (Fig. 4c and e). To further investigate the VEGF/VEGFR2 signaling pathway that mediated downstream STAT3 expression that emerges as a critical transcriptional factor for numerous angiogenesis-associated molecules, we examined the expression of total and phosphorylated STAT3 in HUVECs treated with alkaloid extract of YHS. We demonstrated that the STAT3 total expression level remained unchanged following treatment, whereas STAT3 phosphorylation was limited by alkaloid extract of YHS in a concentration-dependent manner (Fig. 4c and f). The activity of STAT3 was also modulated by subcellular localization, we thus attempted to explore the subcellular distribution of STAT3 by immunofluorescence staining. As illustrated in Fig. 4g, HUVECs cells treatment with alkaloid extract of YHS (from 10 to 100 μg/ml) under VEGF markedly decreased broad nuclear translocation of STAT3, while VEGF rendered STAT3 stability and general nuclear distribution.

Given the fact that p38MAPK and c-Jun N-terminal kinase cascades (JNK) are also the major signaling pathways that modulate various functions of endothelial cells in response to VEGF [34, 35], the effects of alkaloid extract of YHS on these two signaling pathways were assessed. It was observed that there were no obvious changes in both phosphorylation and total expression of P38 (Additional file 1: Fig. S1A and S1B) and JNK (Additional file 1: Fig. S1C and S1D) after the stimulation of VEGF in the presence of various concentrations of alkaloid extract of YHS (from 10 to 100 μg/ml). All of these results indicated that alkaloid extract of YHS inhibited angiogenesis through direct inhibition of VEGFR2 on the surface of endothelial cells, and this inhibitory effect was transduced through the phosphorylation of three key mediators AKT, ERK and STAT3, which are all known as classic VEGFR2 downstream molecules.

## Discussion

Angiogenesis serves a fundamental role in the pathogenesis of angiogenesis-related diseases such as tumor progression, diabetic retinopathy and age-related macular degeneration, thus anti-angiogenesis has been recognized as an efficient strategy for the treatment of these diseases [36]. Among the numerous molecular elements contributing to the angiogenic process, VEGF/VEGFR-2 signaling pathway appears to play a critical role in mediating cellular responses involved in angiogenesis prominently [37]. In clinic, various orally active small molecular inhibitors of VEGFR-2 tend to cause distinct adverse effects though they produce striking benefits for patients with angiogenesis-associated diseases [38]. Therefore, discovery of a novel VEGFR2 inhibitor that is affordable, safe and effective will bring in great potential. It has been recognized that Traditional Chinese Medicine (TCM) acts as a potential source of new drug candidates, thus it can be a very good idea to identify novel VEGFR2 inhibitors from TCM.

In this present study, we illustrated that alkaloid extract of YHS displayed striking inhibitory effect on VEGF-induced angiogenesis via both in vitro and in vivo models. In light of proliferation of endothelial cells, alkaloid extract of YHS suppressed HUVEC proliferation in a concentration- and time-dependent manner by using MTS assay and Ki67 immunofluorescence staining, it was also observed that alkaloid extract of YHS performed better in response to VEGF, suggesting extracellular VEGF emerged as a strong attractant and VEGF-induced signaling pathway contributed to the inhibitory effects of alkaloid extract of YHS on endothelial cell proliferation. Notably, this inhibitory effect of alkaloid extract of YHS was not possibly attributed to cytotoxicity at the cellular level because no obvious toxicity was found at the working concentrations in LDH assay. Moreover, it has been reported that endothelial cell migration driven by VEGF is an essential step of angiogenesis [4]. In this study, we elucidated that both HUVEC horizontal and vertical migration was suppressed by alkaloid extract of YHS in a dose-dependent manner. This might be related to cytoskeleton, which is integral to the modulation of cellular movement. Of note, the three dimensional (3D) environment within a whole organism is much more complicated, and endothelial cells need to move among other cells while they interpret attractive and repulsive cues to choose their path [39]. Hence, 3D co-culture on endothelial cells with basement membrane matrix and other cell types is supposed to provide us the potential to mimic the in vivo environment and thus detect crosstalk among cells.

In the process of angiogenesis, sprouting and tube formation are two steps that are both highly dependent on the migration of endothelial cells [40]. Our data demonstrated that alkaloid extract of YHS limited these two critical behaviors of endothelial cells dose-dependently, further confirming its remarkable inhibitory function on angiogenesis. These in vitro findings were also verified in CAM and Matrigel plug models, which both uncovered that vascular network formation was dramatically diminished in response to alkaloid extract of YHS. Since tumor progression is a classic disease involving pathological angiogenesis, we can next examine the potential effect of alkaloid extract of YHS on tumor angiogenesis. This supporting evidence might provide an experimental basis for further study of YHS in safe clinical application as a potent angiogenesis inhibitor.

Further intrinsic mechanisms for the above anti-angiogenic activities of alkaloid extract of YHS verified that the activation of VEGF/VEGFR2 signaling pathway in HUVECs was inhibited. VEGF has been shown to play a central role in angiogenesis and cell survival pathways, and has been demonstrated to bind to VEGFR2 leading to phosphorylation of Tyr1175 and triggering a signaling cascade contributing to angiogenesis, permeability or survival [41, 42]. Our data demonstrated that alkaloid extract of YHS was able to block Tyr1175 phosphorylation of VEGFR-2 induced by VEGF. It has also been documented that numerous VEGFR-2 downstream signaling mediators such as AKT, ERK, JNK, STAT3 and eNOS were also involved in regulation of endothelial cells survival and proliferation. In particular, SRC/AKT, PKC/ERK and JAK2/STAT3 signalings have been identified as potent cascades in angiogenesis. We illustrated that treatment with alkaloid extract of YHS showed sharp decreases in the phosphorylation of AKT, ERK and STAT3 while did not result in the changes of their total levels, suggesting that alkaloid extract of YHS blocked angiogenesis by inhibiting VEGFR2 and suppressing its downstream signaling components. Surprisingly, translocation of transcriptional factor STAT3 from cytoplasm into nucleus stimulated by VEGF, a pivotal process for inducing expression of a series of angiogenesis-associated genes including ANG2 and COX-2 [43], was blocked by alkaloid extract of YHS in a concentration-dependent manner. This indicates the inhibitory effect on VEGFR2 may be amplified via limiting STAT3 to activate more angiogenic factors. In addition, p38 and JNK are another two MAP kinases that mediate signal transduction from cell surface receptors to the nucleus, regulating programmed cell death by modulating a couple of anti-apoptotic proteins and influencing cell cycle via affecting some cell cycle associated proteins [44]. Our data revealed that both p38 and JNK signaling pathways did not respond to the treatment of alkaloid extract of YHS, indicating that the decreased activation of VEGFR2 by alkaloid extract of YHS subsequently attenuated the activation of SRC/AKT, PKC/ERK and JAK2/STAT3 signalings rather than p38 and JNK MAP kinases signalings.

## Conclusion

**Fig. 5:**
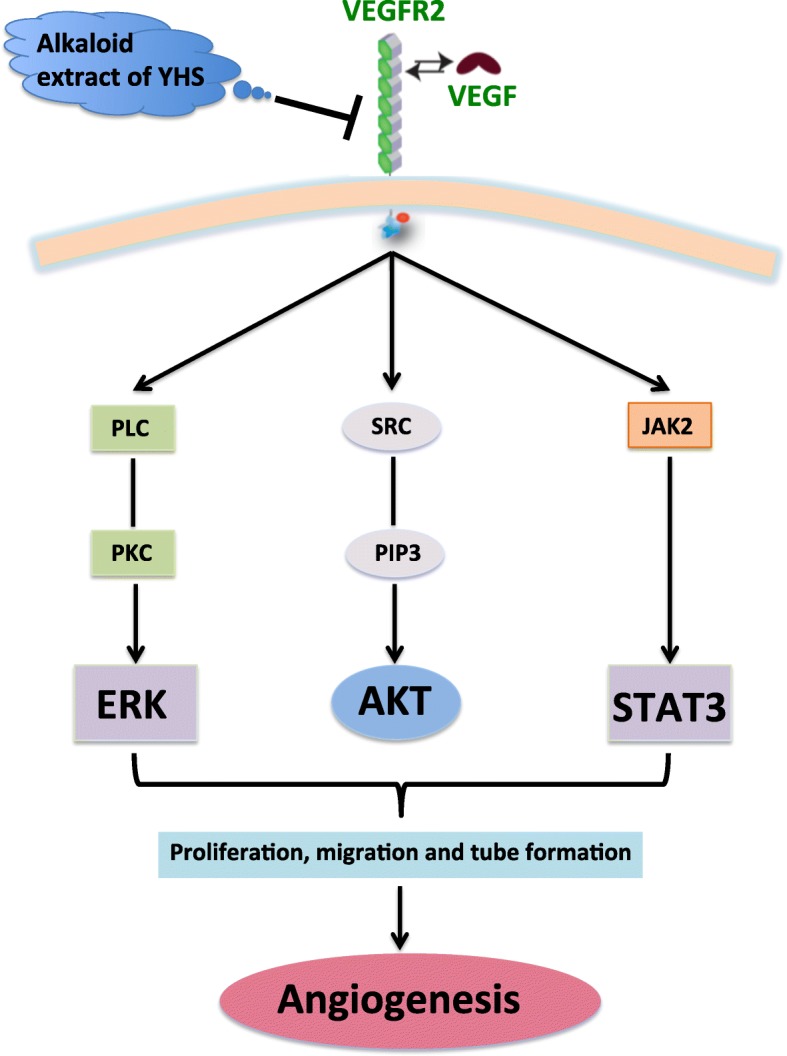
Schematic diagram of effects of Alkaloid extract of YHS on Angiogenesis Alkaloid extract of YHS exerted anti-angiogenic effect on HUVECs by attenuating the activation of VEGF/VEGFR2 signaling pathway and its downstream mediators including AKT, ERK and STAT3. The blocked VEGF/VEGFR2 signaling by alkaloid extract of YHS lead to decreased proliferation, migration and tube formation of endothelial cells

Taken together, our study presents experimental evidence strongly revealing the significant role of alkaloid extract of YHS in preventing angiogenesis both in vitro and in vivo. Alkaloid extract of YHS treatment not only diminished the proliferation, migration, sprouting and tube formation of endothelial cells, but also repressed the blood vessel formation in both CAM and Matrigel Plug models. The underlying mechanism of inhibited angiogenesis by alkaloid extract of YHS was due to that the activation of VEGFR2 and its downstream mediators AKT, ERK and STAT3 were abrogated (Fig. 5). Therefore, this study provided insights into the angiogenesis process by using different models as well as highlighted alkaloid extract of YHS’s anti-angiogenic effect via disrupting VEGF/VEGFR2 signaling pathway, which likely supply health benefits for patients with angiogenesis-associated diseases.

## Supplementary information

**Additional file 1: Figure S1.** Alkaloid extract of YHS had no obvious effects on phosphorylation of p38 and JNK (A) Western blot bands for phosphorylation of p38 and total p38 in HUVECs lysates 24 h following various concentrations of alkaloid extract of YHS treatment in the presence of VEGF. β-actin was used as a loading control. (B) Densitometric ratios for p38 activities were quantified. Data are presented as the normalized expression of mean of three independent HUVEC lines ± SEM, paired t-test. (C) Western blot bands for phosphorylation of JNK and total JNK in HUVECs lysates 24 h following various concentrations of alkaloid extract of YHS treatment in the presence of VEGF. β-actin was used as a loading control. (D) Densitometric ratios for JNK activities were quantified. Data are presented as the normalized expression of mean of three independent HUVEC lines ± SEM, paired t-test.

## Data Availability

The data presented in this study are contained and described within the article, and are available from the corresponding authors upon reasonable request. All materials used in this study are properly included in Methods section.

## Abbreviations

BAD: BCL-2 associated death promoter
CAM: chick embryo chorioallantoic membrane
CYPs: cytochromes P450
EBSS: Earle’s balanced salt solution
ECL: enhanced chemiluminescence
ERK: extracellular signal-regulated kinase
FDA: Food and Drug Administration
HUVECs: human umbilical vein endothelial cells
JNK: c-Jun N-terminal kinase
LDH: lactate dehydrogenase
MAPK: mitogen-activated protein kinase
MTT: 3-(4,5-dimethylthiazol-2-yl)-2,5-diphenyltetrazolium
PDK: phosphoinositide-dependent kinases
STAT3: signal transducer and activator of transcription
TCM: Traditional Chinese Medicine
VEGF: vascular endothelial growth factor
YHS: *Corydalis yanhusuo* W.T. Wang
