# Supplementary material for: Alkaloid extract of Corydalis yanhusuo inhibits angiogenesis via targeting vascular endothelial growth factor receptor signaling
Source: BMC Complement Altern Med. 2019 Dec 10;19:359. doi: 10.1186/s12906-019-2739-6 (PMC6905101; doi:10.1186/s12906-019-2739-6)
Supplement: Supplementary file 1 — Additional file 1: Figure S1. Alkaloid extract of YHS had no obvious effects on phosphorylation of p38 and JNK (A) Western blot bands for phosphorylation of p38 and total p38 in HUVECs lysates 24 h following various concentrations of alkaloid extract of YHS treatment in the presence of VEGF. β-actin was used as a loading control. (B) Densitometric ratios for p38 activities were quantified. Data are presented as the normalized expression of mean of three independent HUVEC lines ± SEM, paired t-test. (C) Western blot bands for phosphorylation of JNK and total JNK in HUVECs lysates 24 h following various concentrations of alkaloid extract of YHS treatment in the presence of VEGF. β-actin was used as a loading control. (D) Densitometric ratios for JNK activities were quantified. Data are presented as the normalized expression of mean of three independent HUVEC lines ± SEM, paired t-test. [file 12906_2019_2739_MOESM1_ESM.pdf]

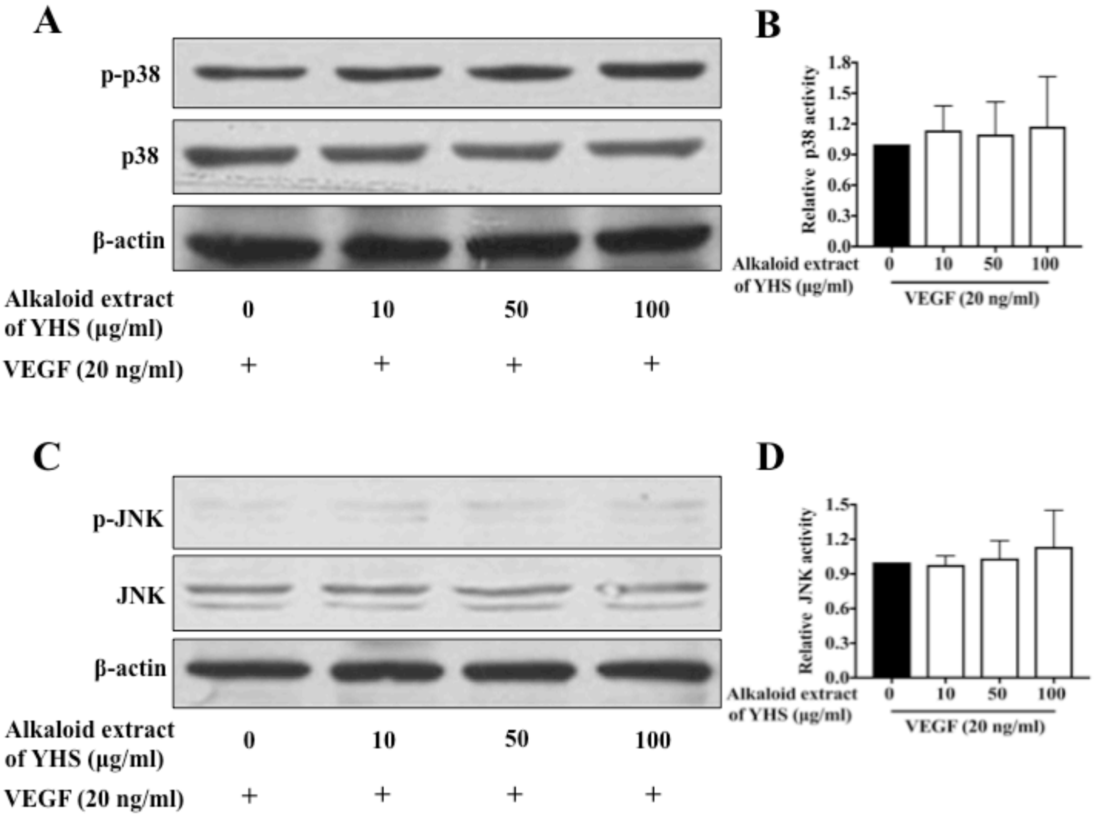

Figure S1. Alkaloid extract of YHS had no obvious effects on phosphorylation of p38 and JNK

(A) Western blot bands for phosphorylation of p38 and total p38 in HUVECs lysates 24 h following various concentrations of alkaloid extract of YHS treatment in the presence of VEGF. β-actin was used as a loading control. (B) Densitometric ratios for p38 activities were quantified. Data were presented as the normalized expression of mean of three independent HUVEC lines ± SEM, paired t-test. (C) Western blot bands for phosphorylation of JNK and total JNK in HUVECs lysates 24 h following various concentrations of alkaloid extract of YHS treatment in the presence of VEGF. β-actin was used as a loading control. (D) Densitometric ratios for JNK activities

were quantified. Data were presented as the normalized expression of mean of three independent HUVEC lines ± SEM, paired t-test.

Matrigel plug model			CAM model		
10 µg/ml	50 µg/ml	100 µg/ml	10 µg/ml	50 µg/ml	100 µg/ml
3.5±5.5	29.2±8.2*	45.3±5.9***	10.5±4*	29.5±8.5*	45.3±3.9***

Table 1. The percent inhibition of angiogenesis (Mean ± SEM, %)
